# The Frames of Reference of the Motor-Visual Aftereffect

**DOI:** 10.1371/journal.pone.0040892

**Published:** 2012-07-23

**Authors:** Guido Barchiesi, Susan Wache, Luigi Cattaneo

**Affiliations:** Center for Mind/Brain Sciences – CIMeC – University of Trento, Trento, Italy; University of Bologna, Italy

## Abstract

Repeatedly performing similar motor acts produces short-term adaptive changes in the agent’s motor system. One striking use-dependent effect is the motor-to-visual aftereffect (MVA), a short-lasting negative bias in the conceptual categorization of visually-presented training-related motor behavior. The MVA is considered the behavioral counterpart of the adaptation of visuomotor neurons that code for congruent executed and observed motor acts. Here we characterize which features of the motor training generate the MVA, along 3 main dimensions: a) the relative role of motor acts vs. the semantics of the task-set; b) the role of muscular-specific vs. goal-specific training and c) the spatial frame of reference with respect to the whole body. Participants were asked to repeatedly push or pull some small objects in a bowl as we varied different components of adapting actions across three experiments. The results show that a) the semantic value of the instructions given to the participant have no role in generating the MVA, which depends only on the motor meaning of the training act; b) both intrinsic body movements and extrinsic action goals contribute simultaneously to the genesis of the MVA and c) changes in the relative position of the acting hand compared to the observed hand, when they do not involve changes to the movement performed or to the action meaning, do not have an effect on the MVA. In these series of experiments we confirm that recent motor experiences produce measurable changes in how humans see each others’ actions. The MVA is an exquisite motor effect generated by two distinct motor sub-systems, one operating in an intrinsic, muscular specific, frame of reference and the other operating in an extrinsic motor space.

## Introduction

A novel procedure for investigating in healthy humans the contribution of the motor system to cognitive processes has been recently developed on the basic assumption of use-induced motor plasticity. This consists in fast changes in the cortical representation of selected movements that occur when such movements are repeatedly performed [1]. In several experiments we have used such phenomenon to induce predictable and selective changes in the representation of given actions in the motor system and to test the consequences of such intervention on the processing of linguistic [2], phonological [3] or visual [4], [5] stimuli. It is by using this approach that we described the so-called motor-visual after-effect (MVA), a phenomenon occurring in healthy adult individuals consisting in a short-lasting change in the way they categorize others’ behavior that is produced by repeatedly performing a given action. As in an adaptation-like phenomenon, the categorization bias consists in a disadvantage in processing the action that has been trained. In particular, in our original description of the phenomenon [5], the performance of the action of “pushing away” objects from oneself produced a disadvantage in interpreting others’ actions as “pushing away”. Vice-versa, repeatedly “pulling towards” oneself some small objects produced a disadvantage in categorizing others’ actions as “pulling”. Interestingly, we showed with event-related transcranial magnetic stimulation (TMS) that single TMS pulses on the left ventral precentral region (ventral premotor cortex-PMv) selectively impaired the adaptation effect. This was taken as evidence, according to the expectations of a TMS-adaptation paradigm [6], of the presence of adaptable visuo-motor neurons coding for both executed and observed actions in the left PMv.

A second, novel aspect to this methodology is that it is based on the induction of plastic changes in the representation of motor acts rather than of simple movements as in the original descriptions of use-induced motor plasticity (in which simple non-goal-directed thumb movements were practiced [1], [7], [8], [9]). We define as motor act a behavioral element that is hierarchically superior to simple joint displacements and is defined by its specific goal [10]. For example, the act of grasping, irrespective of all its kinematic variants, can be defined as a motor act. In all our previous attempts to modulate the motor cortical memory with training, we employed training procedures based on the repetition of goal-directed motor acts, such as grasping and placing [2], [4] or pushing [5] with the upper limb or performing orofacial gestures [3]. Interestingly we found that also the dimension of the motor goal can be subject to plastic use-dependent changes. Indeed all the practiced motor acts consisted in motor acts that contained alternating symmetrical movements (such as flexing and extending the wrist when repeatedly moving small objects with the fingertips) in which only the hand-object relation defined the goal of the motor act (if the hand touched the object with the dorsal surface of the fingers, it becomes a “pushing-away” motor act, but if the hand makes contact with the objects with the palmar surface it becomes a “move-closer” motor act). The cognitive aftereffects that we measured were all related to the goal of the practiced motor acts, in spite of the fact that opposite motor acts (e.g. pushing and pulling) were achieved with very similar sequences of simple movements.

**Figure 1:**
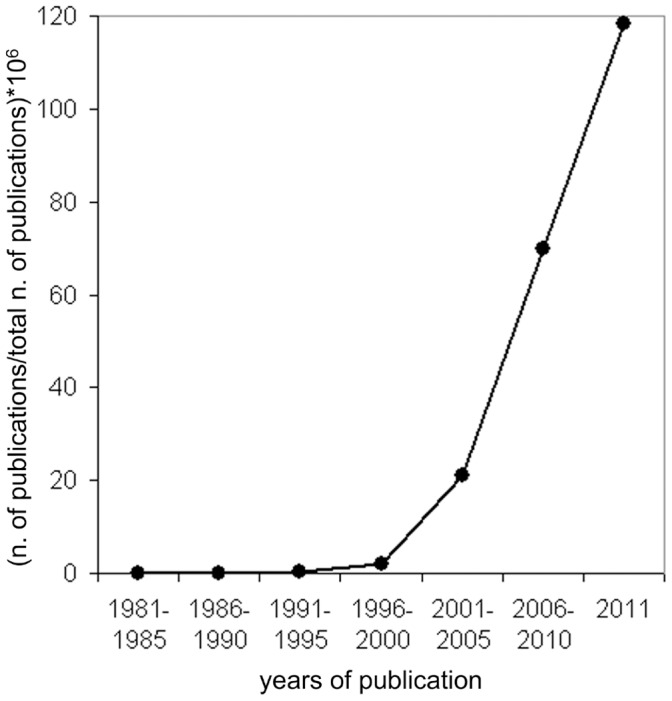
Publications on the topic of action observation in the last 3 decades. The ncbi/PubMed database has been searched for the phrases “action observation” OR “action understanding” in the indicated time-intervals. The resulting number of records has been normalized by the total number of publications indexed in PubMed in the corresponding time-interval. The result has been multiplied by 10^Λ^6 for plotting convenience.

The main interest arising from the description of the MVA and the investigation on its physiological bases is that the MVA represents causal evidence of a role of the motor system in action categorization in healthy humans. Indeed one theory on how the brain represents its environment assumes that categorical knowledge is at least in part stored as a sensorimotor representation specifying how the body commonly interacts with a category [11]. The representation of others’ actions in the brain has been a highly controversial topic since the discovery of mirror neurons (MNs) in the monkey ventral premotor cortex. MNs are multimodal sensorimotor neurons firing when the monkey performs a motor act and when the same act is observed. Their discovery resulted in a ‘hardcore’ version of the embodied cognition of actions, based on the idea of ‘direct matching’ between the sensory experience of others’ actions and the observer’s own motor system. This hypothesis fuelled an ever-growing debate on action understanding, a topic that had previously been neglected by the cognitive neurosciences (see Figure 1). At present it is still a matter of discussion whether the perceptual or conceptual representations of actions require the engagement of the motor system. The evidence in favor of the participation of own movement in action cognition leads to clear-cut implications for the MN-based hypothesis. One is that the perception of an action should activate a corresponding motor program in the human observer. By now a wide range of empirical findings in the field of psychology, neurophysiology and neuroimaging have supported this prediction (for a review see [10]). The second implication is that changes in the activity of the observer’s motor system should affect the way in which others’ actions are categorized. Evidence for this contention is less represented in the literature. Some of this evidence comes from neuropsychological findings with apraxic patients. For example in one group of apraxic patients an effector-specific association was found between deficits in recognition of action sounds and deficits in producing the actions corresponding to that sound [12]. Other authors found a clear association between production and understanding of gestures at the group level for apraxic patients, although in one single subject a dissociation between the observation and execution was evident [13]. In a more recent study using Voxel-based Lesion-Symptom Mapping analyses, deficits in action understanding were mapped, among others, to typically visuo-motor areas such as the premotor cortex [14]. Other evidence for a motor-to-conceptual influence in action understanding is purely behavioral, showing that action performance simultaneous with action observation produces a compatibility-effect on the categorization of the latter (for a review see [15]). Transcranial magnetic stimulation studies have provided indirect evidence for motor-conceptual effects by inducing ‘virtual lesions’ in premotor areas which in turn produced deficits in action categorization, such as disrupted judgment of action kinematics (as in [16]) or alteration of neurophysiological measures related to the implicit distinction between possible and impossible actions (as in [17]). To this respect it is of great interest to mention the results of a recent study [18] in which the consolidation of visuo-motor memories in the motor cortex was effectively inhibited by repetitive TMS over the PMv region.

The MVA needs to be better characterized in terms of what features of the adapting task are effective in producing it and in what spatial frame of reference the adaptation occurs. In the present work we try to characterize the MVA according to 3 distinct dimensions. First we assessed the role of the semantic aspects of the task and instructions. Indeed one alternative explanation of the MVA is that the adaptation is not produced by motor training but rather results from lexical-semantic satiation arising from rehearsal of the instruction during the motor training. In this case the phenomena giving rise to the MVA would all be restricted to the conceptual system, involving no cross-talk with the motor system. In order to exclude this possibility we ran the same experiment as in [5] while reversing the relationship between the instruction and the motor act. In order to do this a puppet was placed on the other side of the bowl, facing the participant: the instructions remained the same as in [5], but now referred to the puppet, and since the puppet was placed on the other side of the bowl with respect to the participant, the instruction “far” corresponded to the same motor act as the instruction “near” in [5] and vice versa. If adaptation is caused by lexical rehearsal, then the “far from the puppet” training should lead to an MVA centered on the instruction’s object (the agent in [5] but the puppet in the present experiment). Otherwise if the adaptation is a truly motor one, then the pattern should depend on the direction of movement and therefore, given the same instruction semantics, a direction opposite to that in [5]. The results clearly showed that the instruction semantics are irrelevant to the production of the MVA.

Second we tried to define the spatial frame of reference in which the adaptation phenomenon takes place. The main distinction we tried to capture was that between “intrinsic” and “extrinsic” motor space. This distinction comes from studies in primate neurophysiology of visuomotor neurons in the parietal, premotor and motor cortex. [19], [20], [21], [22] Some of these neurons can code the spatial end-position of movement in body-centered coordinates. For example a neuron that fires to produce a clockwise rotation of the wrist independently of the initial wrist position will produce movements with different endpoints. Such visuomotor neurons are located in an “intrinsic” spatial frame of reference. On the contrary, some neurons fire to produce any type of movement that has the same target, irrespective of the initial position of the body part. These neurons can produce a range of different wrist movements as long as the spatial position of the target is the same. The frame of reference of such neurons is said to be “extrinsic”. Are the movements generating the MVA mapped in a motor-invariant frame of reference or in an action-invariant (in this case spatial-invariant) frame of reference? To answer this question we performed the second experiment of the present series, in which we manipulated the paradigm so that different movements were used to achieve the same goal (spatial endpoint of the movement). In particular participants were required either A) to use the same movements as in the target pictures (pushing with the dorsum of the hand and pulling with the palmar surface of the hand) to achieve the same goals or B) to use the movements opposite to those in the target pictures in order to achieve the same goals (pushing with the palm of the hand and pulling with the dorsum). Our findings showed that both spatial goal AND movement have an effect in producing the MVA, thus indicating that two distinct neural systems (one operating in intrinsic and the other in an extrinsic motor space) are engaged by the training.

In the last experiment we explored the role of spatial compatibility between the observed action and the participant’s hand posture. We therefore replicated the experiment with the standard instructions but participants were required to orient their hand rightwards or leftwards while acting on the objects and these two conditions were factorially associated with the leftwards or rightwards orientation of the hand in the target pictures. Also in this case we replicated the basic finding of the object displacement direction producing the MVA, with no interaction effects with the spatial congruence or incongruence of the targets.

## Materials and Methods

### General Procedure

**Figure 2:**
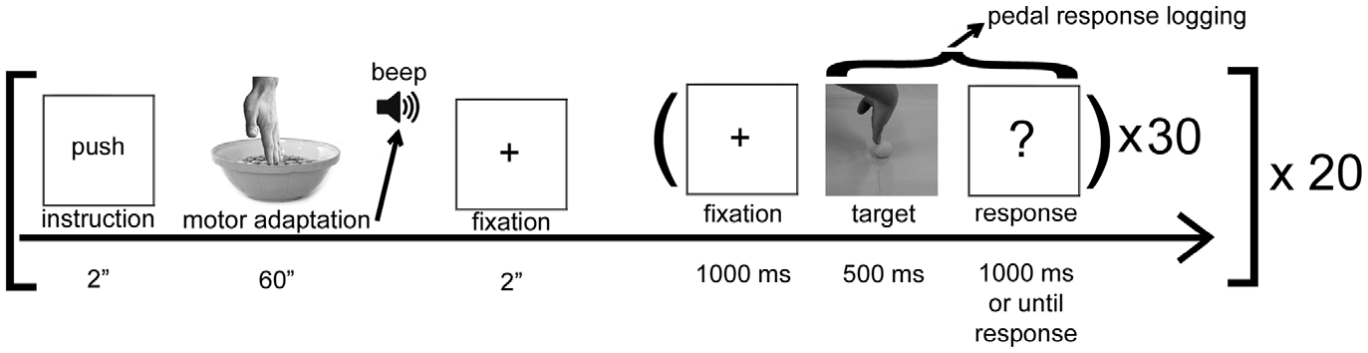
Timeline of the experimental protocol.

Each of the present experiments was organized in series of blocks, and each block comprised two distinct phases. In the first phase the participants performed the adaptation motor task for 60 seconds. In the second phase the participants performed the categorization task on a series of static visual stimuli presented serially. Each target picture was presented for 500 ms. In the previous description of the MVA we found that, consistent with an adaptation effect, the MVA appeared to be waning in the first few trials of the categorization phase (see Figure 2 of [5]). Therefore in every experiment we considered for analysis only the first 15 trials of the categorization phase. An illustration of the general procedures is presented in Figure 2.

### Participants

We tested 16 participants (7 females, age range: 19–37) in the “Semantic reference” experiment, 20 participants (12 females, age range 22–34) in the “inverted effector” experiment, and 20 participants (14 females, age range 19–33) in the “spatial” experiment. According to the standard handedness inventory [23] all subjects were right handed except one in the spatial experiment group. The present study was approved by the local Ethical Committee for human studies and was conducted in compliance with the Helsinki Declaration of 1975, as revised in 2008 [24]. All participants gave written informed consent to the experiment.

### Visual Stimuli

**Figure 3:**
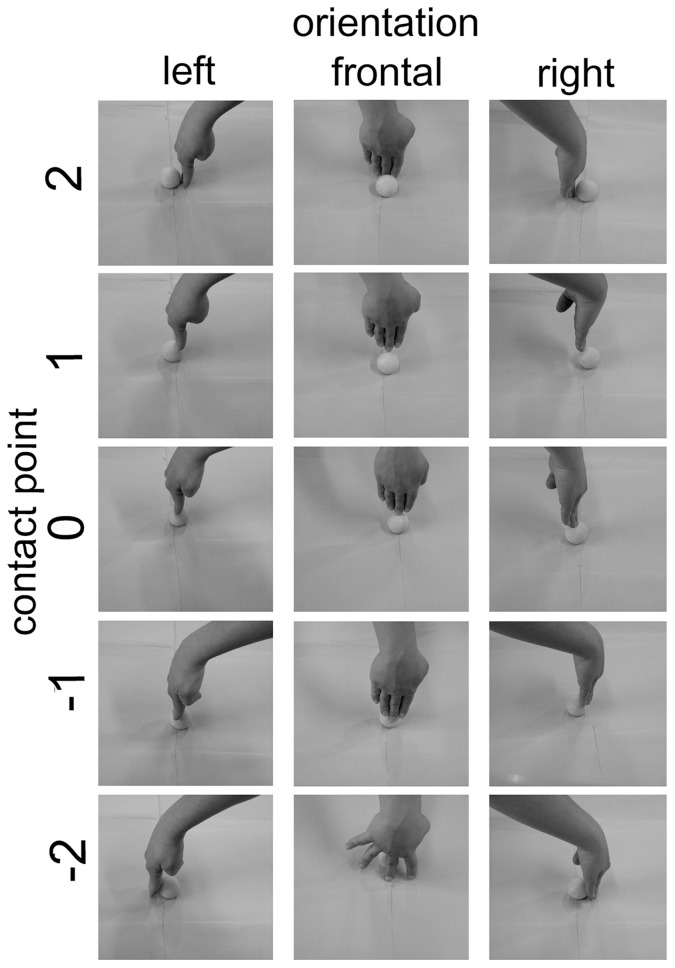
Set of stimuli used in the present experiment. The ambiguous contact point used for analysis is labeled as “0”.

Visual stimuli presented in the categorization phase of the blocks were the same in all 3 experiments. They consisted of 30 pictures of right hands displacing an object (a yellow table-tennis ball). The full set of images is presented in Figure 3. The orientation of the hand in the pictures was 90°, 180°, and −90° with respect to the participant’s viewpoint. The contact point with the object (the table-tennis ball) was at 5 different points including one on the upper pole of the sphere. The remaining 4 were symmetrically placed along a meridian at 20° and 60° from the vertical axis. The hands were all positioned at 90° with respect to the horizontal plane on which the object was positioned. In half of the figures hands touching objects were female hands and in the other half they were male hands balanced across orientation and position. The background of the figures was homogeneous. Stimuli were presented with the E-prime software (Psychology Software Tools Inc.) on a 60 Hz computer screen, with a visual angle of 14° vertically and 19° horizontally.

### Motor Adaptation Procedure

**Figure 4:**
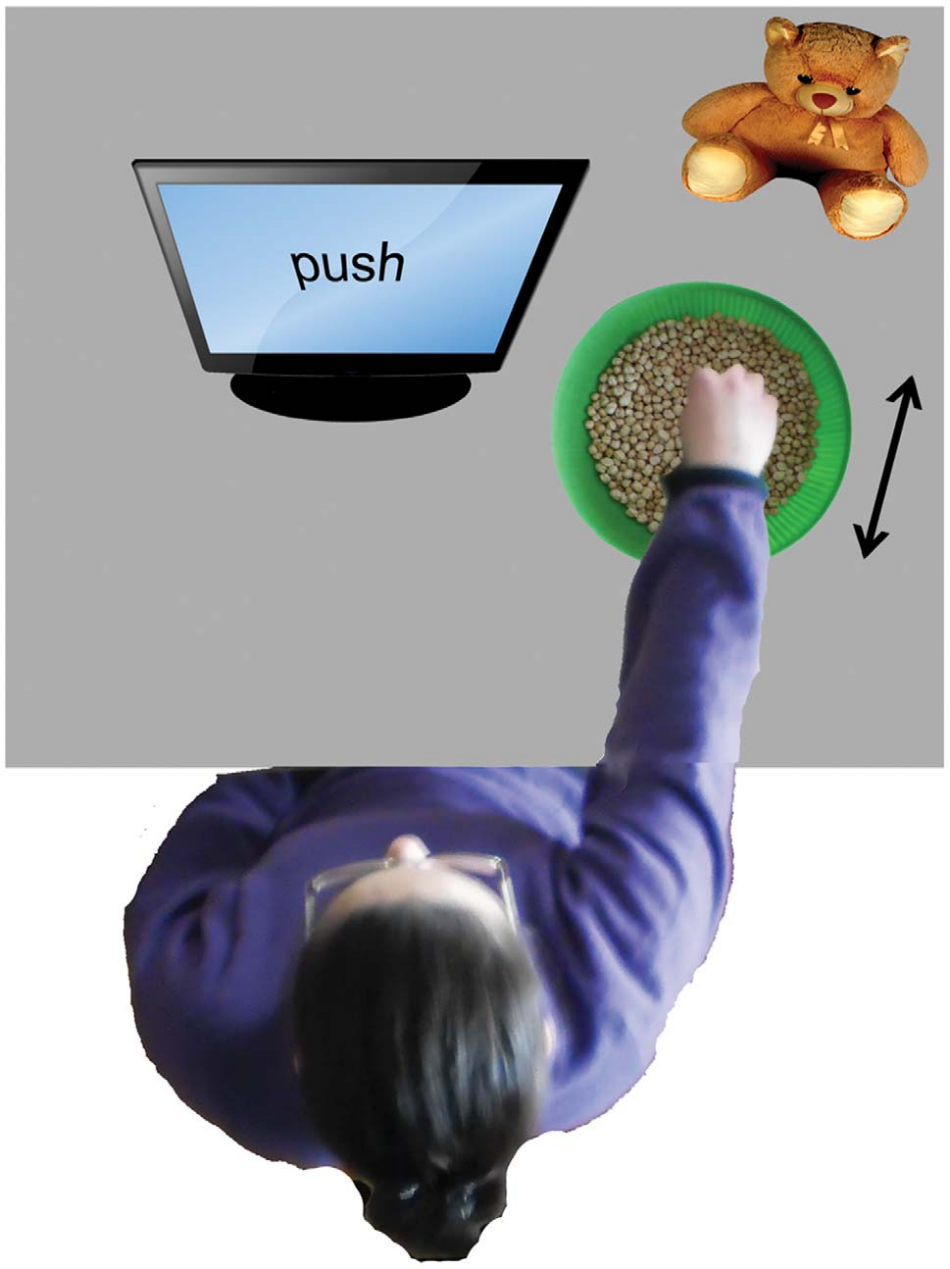
Schematized setup of the “semantic reference” experiment. The arrow indicates the direction of chickpeas displacement.

**Figure 5:**
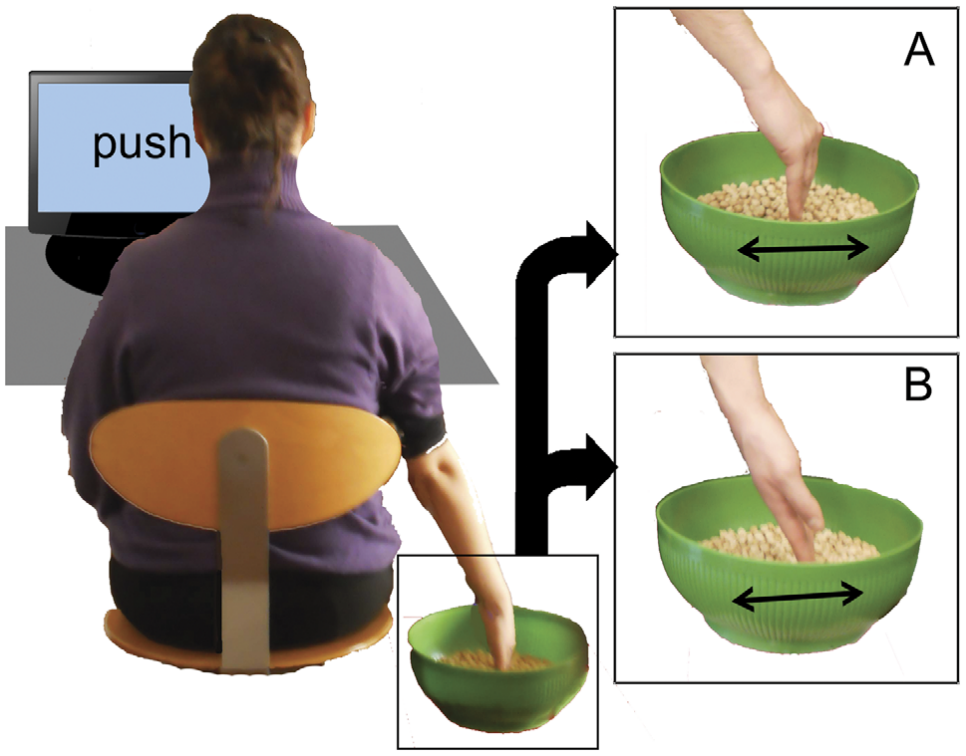
Schematized setup of the “inverted effector” experiment. The arrow indicates the direction of chickpeas displacement.

**Figure 6:**
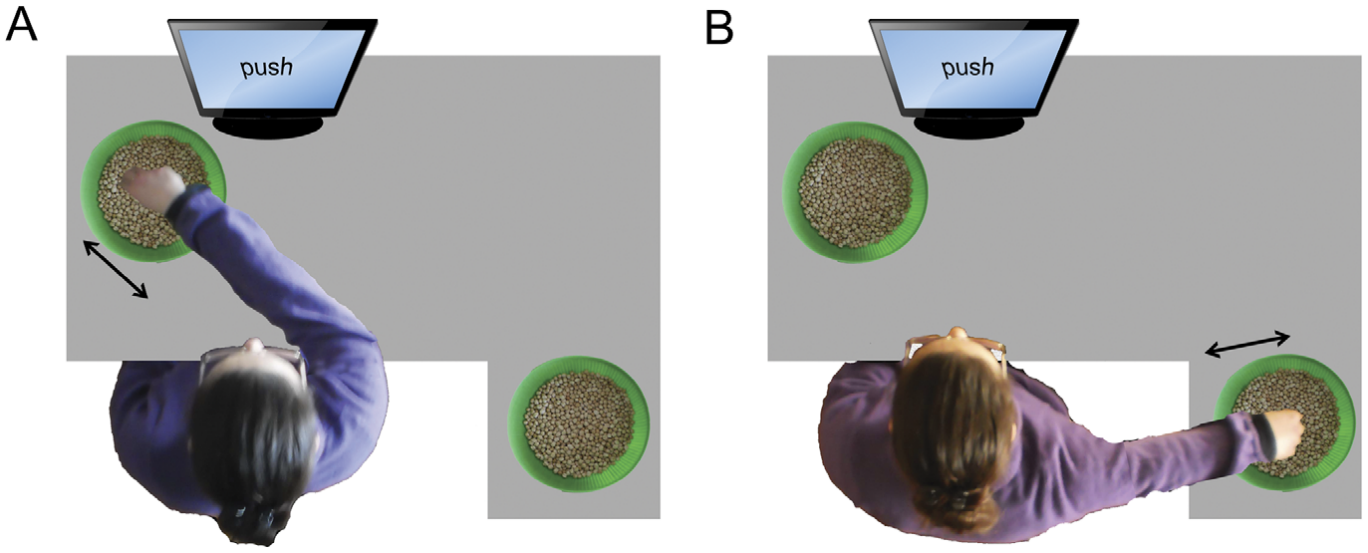
Schematized setup of the “spatial compatibility” experiment. The arrow indicates the direction of chickpeas displacement.

Participants sat comfortably on a chair in front of a computer screen. The objects upon which the participants were asked to act consisted of around 50 dried chickpeas inside a spherical bowl. We used dried vegetables in order to have a series of small, eventually replaceable, objects of uniform size and light weight. Such objects have previously been used in similar use-dependent plasticity induction protocols [2], [4], [5]. The shape of the bowl allowed the chickpeas to fall back naturally in the center of the bowl after the displacement. In this way the direction of chickpea displacement was univocal for each action. The onset of each block was determined by the participant by pressing the space bar on the keyboard placed in front of them. Every block in each experiment started with an instruction displayed on the computer screen for 2 seconds, describing the action to be performed on the chickpeas during the subsequent adaptation phase. Participants used their right hand to displace the chickpeas according to the different instructions. The bowl was placed in different positions with respect to the participant in each of the experiments. The adaptation phase required continuous acting on the objects for 60 seconds until a beep-sound was emitted by loudspeakers connected to the computer. It should be stressed that in order to avoid any access to visual information concerning their hands’ movements, an opaque screen prevented the participant from seeing his/her own right hand during the whole duration of the motor training. In the *“semantic reference”* experiment the bowl was placed in front of the participants. A puppet was placed on the opposite side of the bowl facing the participant (see Figure 4). The experiment consisted in 20 blocks each of which began with an instruction literally translated as “move near to the puppet” (“avvicina al pupazzo” in Italian) or “move away from the puppet” (“allontana dal pupazzo” in Italian). In this way, the externally referenced “move near” instruction corresponded to a self referenced “push” instruction and participants had to continuously move the chickpeas toward the puppet with the back of their hand. Vice-versa a “move away” instruction prompted the act of pushing the chickpeas away from the puppet with the palm of their hand, corresponding to a self-referenced “pull” instruction. The two different instructions were presented in random order across the block series. In the *“inverted effector”* experiment the bowl was placed on the participant’s right side at a level lower than the table plane (Figure 5). The experiment consisted of 20 blocks. At the start of each block the instruction indicated both the goal of the action and the hand part with which the action had to be performed, resulting in 4 different instructions: “move away from yourself with the back of the hand”, “move away from yourself with the palm of the hand”, “move towards yourself with the back of the hand”, “move towards yourself with the palm of the hand”. Instructions were presented in random order across the blocks. In the *“spatial compatibility”* experiment we tried to make participants perform the push and pull actions using effector positions that were either compatible or incompatible with the target pictures. We therefore presented the target hand pictures with only the right and the left orientations (first and third columns of Figure 3), because the frontal orientation was obviously not reproducible by the participant. Two bowls were simultaneously present in the experimental scene on the computer table, one in front of the subject (from now on defined as Bowl A) and the other one (defined as Bowl B) on his/her right side (Figure 6). In this way the hand acting in the lateral bowl had the same spatial orientation as in the rightward target pictures (in the third column of Figure 3). The participant’s hand acting on the frontal bowl was oriented 30–45° leftwards with respect to the midline. A full 90° rotation was attempted at first but this proved to be very fatiguing for participants. The participant’s hand when acting on this bowl was oriented as in the leftward directed target pictures (first column of Figure 3). The experiment consisted in 20 blocks, half of which were performed with the anterior bowl and the other half with the lateral one. At the onset of each block the instruction that was presented indicated both the direction of object displacement and the bowl to be used. Therefore the 4 different instructions were “move away from yourself in bowl A”; “move towards yourself in bowl A”; “move away from yourself in bowl B” or “move towards yourself in bowl B”. Instructions were presented randomly.

### Categorization Phase

The categorization phase was the same for all 3 experiments. It started 2 seconds after the “beep” sound indicating the end of the motor adaptation phase. Participants were required to quickly take their hand away from the bowl and to put it palm-down on the table. In the first 2 experiments, the presentation comprised 30 target pictures in random order (Figure 2). In the third experiment (“spatial compatibility”) only the hands in the lateral viewpoints were presented (columns 1 and 3 of Figure 3). Participants were required to categorize the action of the depicted agent as “moving the object away from oneself” or “moving the object closer to oneself”. Categorization was required to be referred to the depicted agent and not to the observer. For example, the picture in the middle column of Figure 3, at contact point +2 should be correctly categorized as “moving away”. Responses were given as fast and accurately as possible by pressing two pedals with the right and left feet. The response mapping on the feet pedals was balanced across participants. Each of the 30 trials began with a red cross in the center of the screen, lasting 1000 ms, preceding the presentation of the visual stimulus (lasting 500 ms). A white screen with a central “?” sign appeared after the target and persisted until the participants’ response or until a maximum duration of 1000 ms was reached. This series looped until each of the 30 target pictures were presented (Figure 2). Responses were logged from the onset of the target picture. Responses given after 1500 ms from target onset were not logged.

### Data Analysis

The responses to the stimulus set presented in Figure 3 are distributed following a sigmoid psychometric function [5]. This distribution was also verified for the present data by a fitting procedure on the total data set. To simplify the analysis we decided to further consider only the responses to the ambiguous contact position (represented in the middle column of Figure 3). We built a score indicating the average responses to the ambiguous stimuli by attributing a −1 value to single “pull” responses and a +1 value to single “push” responses. Such single-trial values were then averaged within participants and within conditions to obtain a single score ranging between −1 and +1 indicating alternatively, if negative, a prevalence of pull responses and, if positive, a prevalence of “push” responses. This response score was then used in all further analyses as a dependent variable.

In the “semantic reference” experiment the data were analyzed by means of a t-test for paired data comparing individual response scores between the 2 motor adaptation instructions (either “push” or “pull”). Remember that in this experiment the instruction is the opposite as in the other two experiments, that is, the action associated with the “push” instruction in the “semantic reference” experiment corresponds to the one associated with the “pull” instruction in the other two experiments, as well as in the previous description of the MVA [5]. In the “inverted effector” experiment the response scores were averaged within each of the 2 action directions and within each of the two hand postures used in the motor adaptation, in a 2×2 design, resulting in 4 cells per subject. Response scores were then analyzed by means of a 2-way ANOVA with 2 within-subjects factors: ACTION (2 levels: “centrifugal” and “centripetal”) and EFFECTOR (2 levels: “back” and “palm”). In the “spatial compatibility” experiment the response scores were averaged in a 2×2×2 design, within each of the two action directions, within each of the two bowl positions and within each of the 2 orientations of the target picture (see Figure 3), for a total of 12 cells per subject. The data were then analyzed with a 3-way ANOVA with 3 within-subjects factors: ACTION (2 levels: “centrifugal” and “centripetal”), HAND ORIENTATION (2 levels: front or sideways) and TARGET ORIENTATION (2 levels: “left” and “right”).

## Results

The overall rate of responses that were not logged because they were made after the 1500 ms limit was of 3.3% with no significant differences between experiments. None of the participants was excluded from the analysis.

### “Semantic Reference” Experiment

**Figure 7:**
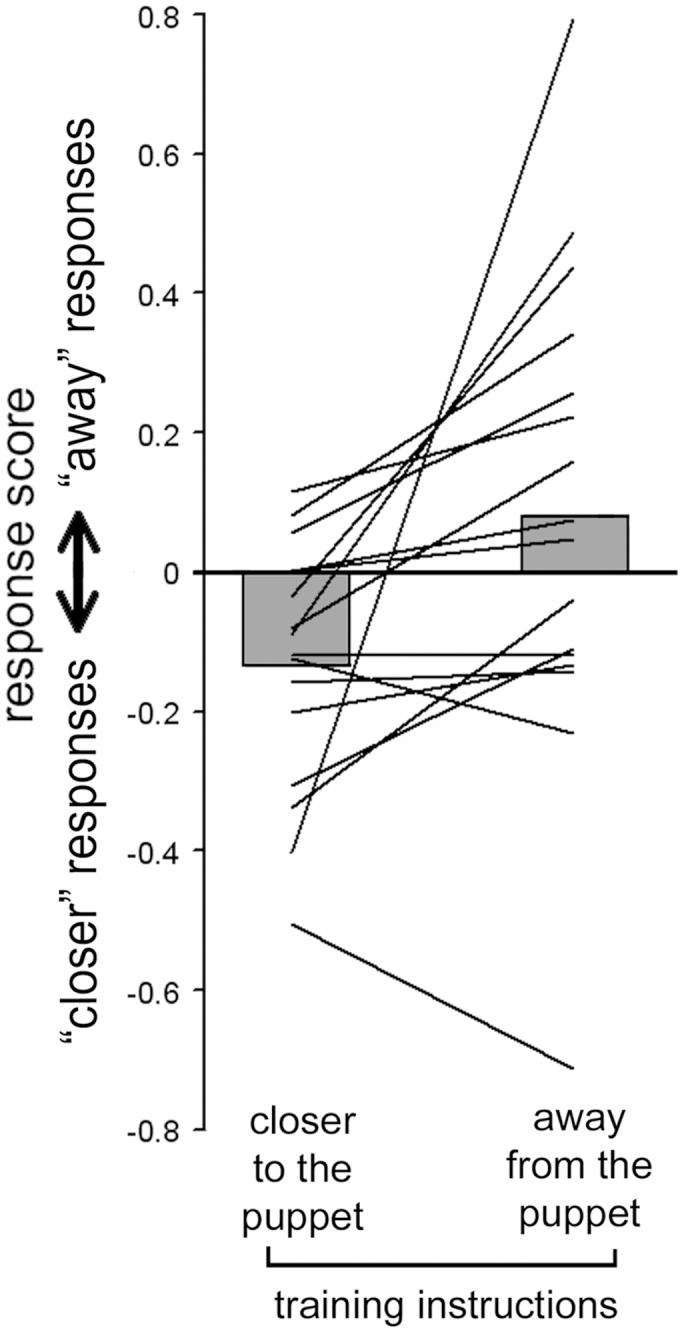
Results of the “semantic reference” experiment. The solid lines represent individual data and the gray bars represent the group averaged values.

The individual and the group results are shown in Figure 7. The t-test showed a significantly different distribution for the paired data (t(15) = −2.61, p = 0.02). The training with displacement of the object towards the puppet, i.e. away from the agent produced significantly more “moving closer” responses (mean score −0.13; SD: 0.18) than the training with displacement of the object away from the puppet, i.e. towards the agent (mean score 0.08; SD: 0.34).

### “Inverted Effector” Experiment

**Figure 8:**
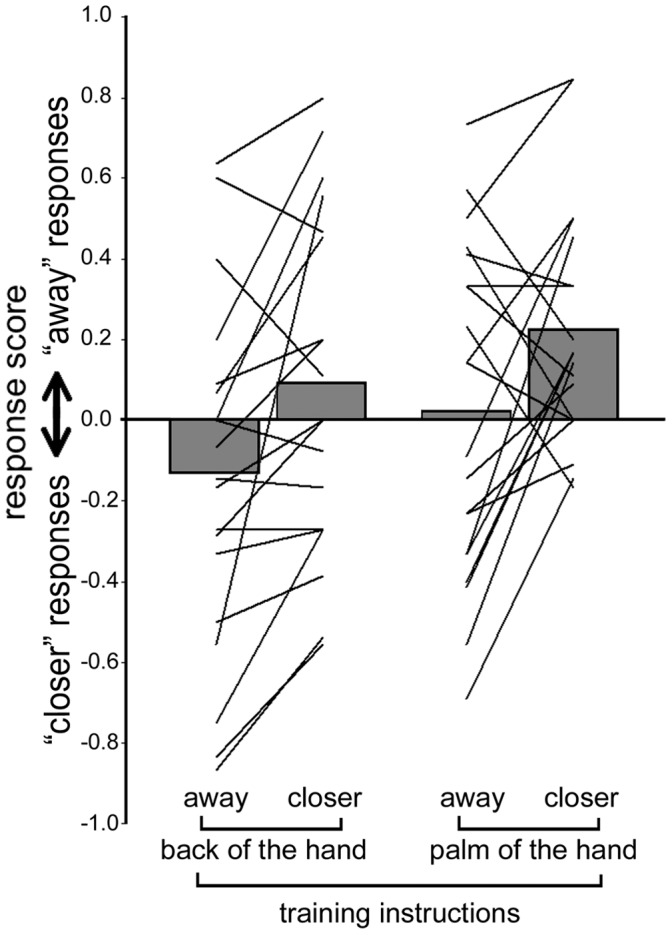
Results of the “inverted effector” experiment. The solid lines represent individual data and the gray bars represent the group averaged values.

The individual and the group results are shown in Figure 8. The ANOVA showed a main effect of both factors: ACTION (F(1, 19) = 6.17, p = 0.02) and EFFECTOR (F(1, 19) = 13.8, p = 0.001). The main effect of ACTION was, again, consistent with our expectations, due to the fact that a repeated displacement of the objects away from the agent produced significantly more “moving closer” responses (mean score: −0.06; SD: 0.42) than the repeated displacement of the objects towards the agent (mean score: 0.15; SD: 0.36). Interestingly, the main effect of EFFECTOR showed that the repeated use of the dorsal surface of the hand produced significantly more “moving closer” responses (i.e. produced a bias in favor of the target picture with the palmar surface of the hand being used; mean score: −0.02; SD: 0.43). By contrast, repeated use of the palmar surface of the hand produced significantly more “moving away” responses (i.e. produced a bias in favor of the target picture with the dorsal surface of the hand being used; mean score: 0.12; SD: 0.37). No interaction between the two factors was present (F(1, 19) = 0.04, p = 0.84).

### “Spatial Compatibility” Experiment

**Figure 9:**
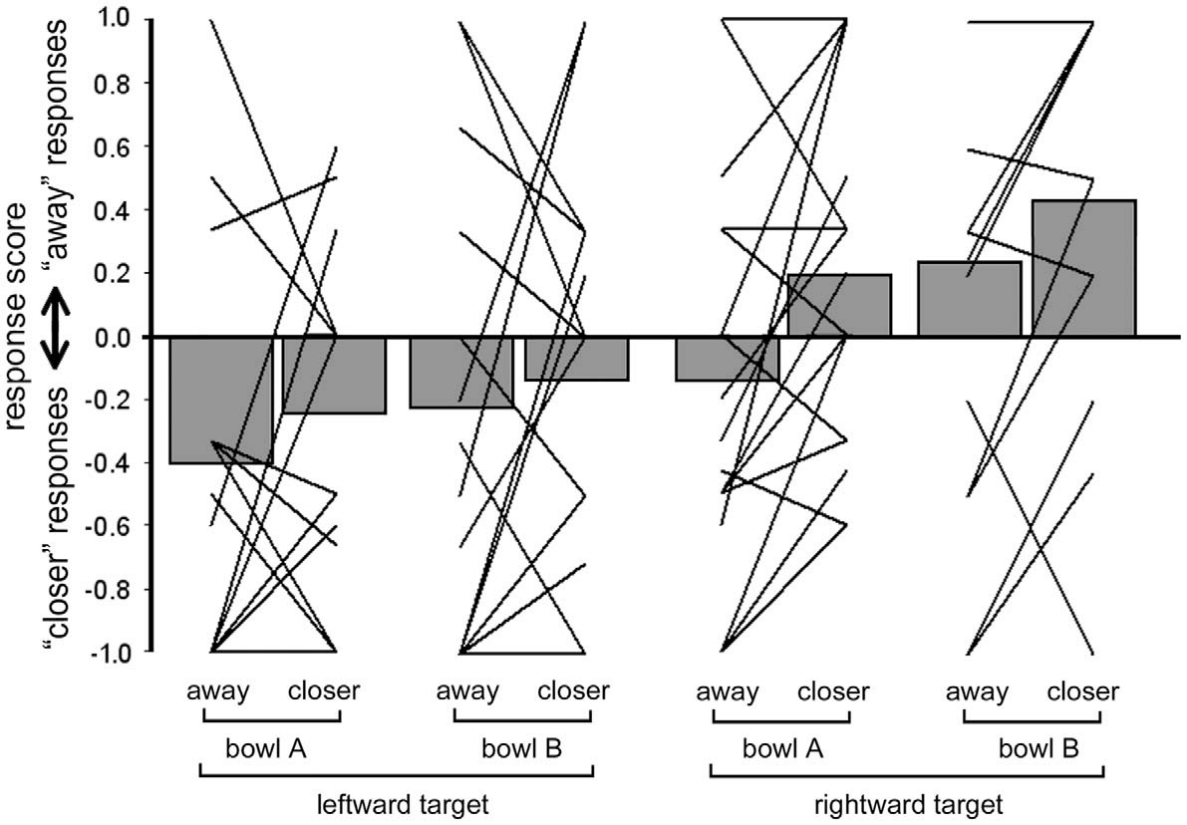
Results of the “spatial compatibility” experiment. The solid lines represent individual data and the gray bars represent the group averaged values.

The individual and the group results are shown in Figure 9. The ANOVA showed a main effect of all 3 factors: ACTION (F(1, 17) = 6.96, p = 0.02), HAND ORIENTATION (F(1, 17) = 5.56, p = 0.03) and TARGET ORIENTATION (F(1, 17) = 9.03, p = 0.008). No interactions between any of the factors were found (all p-values >0.39). The main effect of ACTION consisted, as expected, in a greater rate of “moving closer” responses when the subjects had been pushing the objects away (mean score: −0.16; SD: 0.41) and vice-versa in a greater rate of “moving away” responses when the subjects had been moving closer the objects (mean score: 0.06; SD 0.32). The main effect of HAND ORIENTATION was due to participants responding more frequently “moving closer” to target pictures when they had acted with the hand oriented leftwards, in bowl A (mean score: −0.15; SD: 0.37) and responding more frequently “moving away” when they had acted with the hand oriented rightwards, in bowl B (mean score: 0.05; SD: 0.38). Finally, the main effect of TARGET ORIENTATION consisted in the fact that subjects responded more frequently “moving closer” (mean score: −0.28; SD: 0.42) to pictures showing a hand oriented leftwards (left column of Figure 3) and responded more frequently “moving away” (mean score: 0.18; SD: 0.49) to pictures showing hands directed rightwards (right column of Figure 3).

## Discussion

The present data indicate that the MVA is a robust and replicable phenomenon. In the present experiments the target visual stimuli were kept constant and identical to our previous description of the MVA [5]. In every experiment we instead manipulated the different components of the adapting actions, so that we could identify the single invariant features between the performed actions and observed target pictures that were responsible for the MVA.

In the first experiment we manipulated the semantics of the verbal instructions for the training actions. This experiment was done to control whether semantic satiation, an adaptation phenomenon in which lexical repetition within a semantic category produces a reduced access to that same category [25], [26], [27], [28] could account for the MVA. Hypothetically, if participants performed a sub-vocal rehearsal of the instructions during the motor adaptation task, this phenomenon could account for the observed MVA. Our results however render this hypothesis unlikely because, although the verbal instructions were inverted compared to the original experiment, the factor that produced the MVA was the direction of the movement referred to the agent rather than to the instructions’ semantics. One limitation of the present experiment is that the puppet was not present in the target pictures. In this way it is still possible that an egocentric frame of reference was used also to translate the semantics of the instruction. Another possible problem is that no internal control condition is present where the instruction “push” actually corresponds to a push movement and this does not allow a direct comparison between congruence-incongruent semantics and action features. However, an indirect comparison can be made with the results of the second and third experiments, where the instruction “push” actually corresponded to a push movement.

In the second experiment we investigated whether it was the direction of the motor act or the actual movements of the wrist and finger joints that produced the MVA. We therefore applied a well-established paradigm in which the goal of the motor act (moving closer to or away from the objects) was dissociated from the movements required to accomplish it. This paradigm has been successfully applied in non-human [17], [18] and human [29] primates to study the representations of goals and movements in the motor system. Our results show an effect of both movements and action goals, with no interaction between the two. Therefore the MVA is produced by the adaptation of two distinct components of the motor system, one operating in an intrinsic (movement-related) spatial frame of reference and the other operating in an extrinsic (goal-related) frame of reference. We showed that performing centrifugal acts produced a bias in categorizing the target pictures in favor of a centripetal displacement of the object and vice-versa, therefore, the action goals of moving the objects away or towards the agent, irrespective of the movements used to achieve them, are a causal component of the MVA. However, superimposed on this, we also observed that whenever the dorsum of the hand was used to displace the objects, irrespective of the direction of displacement, a bias was produced in the categorization of target pictures towards the pictures with the palm of the hand making contact with the object and vice-versa. In our opinion the most plausible interpretation of this datum is that the actual joint displacement is causally involved in producing the MVA. This finding is supported by results in non-human primates, in the motor systems of which, the double coding of goals and movements is widespread [15]–[18]. Another interpretation that cannot be ruled out by the present data is that it is the somatosensory rather than the visual information that determines this component of the MVA. According to this hypothesis the adaptation would occur by repeated contact with the dorsal or palmar surface of the fingers during the actual movements and this in turn would produce a bias in the internal representation of the somatosensory consequences of the observed movement.

In the third experiment we explored the role of visual spatial compatibility between the effector performing the adaptation task and the effector shown in the target pictures. The finding of a main effect of action direction replicates once more the finding of an action goal-driven MVA. The most interesting result of this experiment however is a negative finding, i.e. the absence of interactions between the orientation of the acting hand and the orientation of the hand in the target pictures. This datum indicates that the MVA is not generated by matching of purely spatial features between the observers’ hand and the observed hand. On the contrary, it is based on the matching of actions or movements, irrespective of the viewpoint. It is also worth reminding the reader here that in all experiments the vision of the subject’s own hand was prevented by means of an opaque shield. Finally, in the third experiment, the finding of a main effect of target orientation is of little interest, as it is probably due to an imperfect matching between the visual features of the two oppositely oriented stimuli. Also, the finding of an effect for the position of the bowl, resulting in acts directed to the right or to the left is of little speculative interest since it was not the aim of the experiment.

In conclusion, the present series of experiments shows that the MVA is a phenomenon driven purely by motor behavior and not by non-motor lexical/semantic features or by visual compatibility of the agents’ bodies with the observed actions. Importantly we reinforce the notion that the human motor system contains two distinct coding of self-produced and observed motor behavior, one in an intrinsic frame of reference [30], [31] and the other in an extrinsic frame of reference [29].
